# Trend Observations in Home Parenteral Nutrition. Prevalence, Hospitalizations and Costs: Results from a Nationwide Analysis of Health Care Provider Data

**DOI:** 10.3390/nu13103465

**Published:** 2021-09-29

**Authors:** Marcin Folwarski, Stanisław Kłęk, Agnieszka Szlagatys-Sidorkiewicz, Adam Wyszomirski, Michał Brzeziński, Magdalena Skotnicka

**Affiliations:** 1 Department of Clinical Nutrition and Dietetics, Medical University of Gdansk, 80-210 Gdańsk, Poland; 2 Home Enteral and Parenteral Nutrition Unit, General Surgery Department, Nicolaus Copernicus Hospital, 80-803 Gdańsk, Poland; 3 Surgical Oncology Clinic, Maria Sklodowska-Curie National Cancer Institute, 31-501 Kraków, Poland; stanislaw.klek@onkologia.krakow.pl; 4 Department of Pediatrics, Gastroenterology, Allergology and Nutrition, Medical University of Gdańsk, 80-211 Gdańsk, Poland; agnieszka.szlagatys-sidorkiewicz@gumed.edu.pl (A.S.-S.); brzezinski@gumed.edu.pl (M.B.); 5 Department of Adult Neurology, Faculty of Medicine, Medical University of Gdańsk, 80-211 Gdańsk, Poland; adam.wyszomirski@gumed.edu.pl; 6 Department of Commodity Science, Medical University of Gdańsk, 80-211 Gdańsk, Poland; skotnicka@gumed.edu.pl

**Keywords:** nutrition, home parenteral nutrition, prevalence, age distribution, cost analysis

## Abstract

Background: The population of patients on home parenteral nutrition (HPN) worldwide is growing. Since only a few counties provide data from national registries long-term observations are valuable to address this specific area of nutrition support. This study is a nationwide analysis determining the trends in the epidemiology of HPN (prevalence, age distribution, death rates), indications for HPN, causes for hospitalizations, and cost analysis of HPN reimbursement in Poland between 2010–2020. Methods: A retrospective analysis of data obtained from the national health fund (NHF) of Poland on adult patients on HPN. Results: The prevalence of adult patients on HPN in Poland in 2020 was 53.26 per million citizens with a 2.99-fold increase and a growing trend observed from 2010. Significant decrease in the percentage of patients between 18–34, 45–54 and an increase in patients between 65–74 and patients over 75 years old was observed. Trend analysis showed an increase in new patients between 65–74 and a decrease between 35–54. Malnutrition (34.28%), postprocedural disorders of the GI tract (19.61%), intestinal malabsorption/other intestinal diseases (20.41%) and GI obstruction due to cancer (17.36% as primary and 23.16% as secondary diagnosis) were mostly reported as the primary indications for HPN. Cancer patients were mostly gastric, ovarian and colon cancer (34.74%, 17.83% and 12.3%). HPN and total health cost reimbursement increase was 2.6 and 2.57—fold respectively. Costs of HPN and total health care costs in 2020 per patient were € 10,015 and € 16,038, respectively. Overall death risk rate during the first year of nutrition was 0.59 with a significant increase in the observation period *p*-trend < 0.004. A significant increase in the death rate was observed in patients above 75 years old (estimate 1.629, *p*-trend < 0.030). Cancer, infection, malnutrition and GI symptoms were the most common indications for hospitalizations of HPN patients. The rate of patients with a maximal length of HPN of 5 months in 2010 was 54.9% and was growing up to 78.1% in 2020. Conclusions: The prevalence of HPN in Poland is growing. Trends of age distribution show increasing numbers of patients with more advanced age and shorter survival. Costs of HPN are comparable with other European data.

## 1. Introduction

Home parenteral nutrition (HPN) is a life-sustaining therapy for patients with intestinal failure (IF) who require parenteral nutrition outside of the hospital. IF according to the European Society for Clinical Nutrition and Metabolism (ESPEN) is defined as “the reduction of gut function below the minimum necessary for the absorption of macronutrients and/or water and electrolytes, such that intravenous supplementation (IVS) is required to maintain health and/or growth” [1,2]. 

Advancements in medical care and improvements in life status contribute to prolonging life expectancy in developed countries. Life expectancy at the age of 65 rose for women and men by 9 months and 1.5 years per decade in the Swedish population from 1998 to 2017. The trends were observed also in subpopulations of polymorbid patients with myocardial infarction, ischemic stroke, hemorrhagic stroke or cancer (colon, lung, breast). The gap between the survival of a healthy population and patients with civilizational diseases is also decreasing gradually [3]. Polish demographic statistics confirm those trends showing an increase in 3.4 years of life expectancy of 60-year-old citizens from 1960–2019. Improved treatment of cardiovascular diseases, lower tabacco and alcohol consumption, diet, physical activities, health promotion programs and legislation changes are considered as important contributing factors [4,5]. We observe more patients in advanced age with polymorbidity and longer survival. Therefore, population of HPN patients may continue to grow and we can expect more challenging and polymorbid patients in advanced age. Advanced age and a growing number of cancer patients in HPN contribute to the demands of financial resources in the health care system required to cover all medical needs. It is exceedingly difficult to compare data on real costs of treatment between different countries due to various systems of refoundation and organization of the health care system. Patients on HPN often require surgical procedures, advanced medical treatment of complications and cost-consuming oncological treatment. 

The country’s income is associated with the reimbursement for EN and PN in the hospital and in chronic or home settings [6]. Other observations show that reimbursement of PN increased the use of PN in hospital but the evident influence on home nutrition was not confirmed [7]. A recent systematic review on costs of HPN showed that it is very problematic to compare the data between countries due to different methodologies and high heterogeneity of the studies. Most studies included only direct costs and were designed from a health care perspective [8]. Direct healthcare costs (ex. HPN bags, catheters, monitoring, managing of complications) and direct non-healthcare costs (like transportation of HPN bags or consumables) account for the majority of costs in HPN [9]. 

Organizing HPN teams and planning the organization of HPN requires epidemiological data on this population. Only a few countries around the world gather organized information and provide studies based on registries. Long-term observations and trend analysis of HPN populations give a possibility to estimate future perspectives on epidemiology. Since 1998, HPN has been reimbursed in Poland only by the national health fund (NHF), which gives a unique possibility of data analysis of all patients requiring this procedure. We analyzed the data from NHF in a long-term perspective concerning main prevalence data, indications for the procedure, hospitalizations and the national costs of the reimbursement of HPN. 

## 2. Materials and Methods

This study is a retrospective analysis of adult patients on HPN treated in Poland between 1 January 2010 and 31 December 2020. Anonymized data were collected from NHF—the only national health care provider in Poland. Patient epidemiological statistics were anonymized, and it was not possible to identify individual subjects. The study was conducted in accordance with the ethical standards of the Helsinki Declaration. 

### 2.1. HPN Population; Prevalence Data, Hospitalizations and Long-Term Trends

National prevalence trends were analyzed. Yearly data on the total number of patients on HPN, new qualifications, age, sex distribution were collected. Indications for HPN and comorbid diseases described with the International Classification of Diseases (ICD-10 coding) were collected. A total number of hospitalizations, main indications, comorbid diseases and medical procedures (ICD-10 and ICD-9—Diagnosis and Procedure Codes) involved were analyzed. ICD-10 and ICD-9 coding in Poland is assigned by physicians, required in every medical documentation and reported to NHF. Length of nutrition, death rates during first year of HPN with age distribution and the rates of short term (maximum of 5 months) HPN were analyzed. Statistical analysis of trends in the observation period was calculated for: overall population, new qualifications, age distribution, death rates and short term HPN.

### 2.2. Cost Analysis

Analyzed data were obtained from the Polish NHF. Yearly information on total reimbursement of the HPN, total health-related costs of HPN patients. Those data were calculated per one HPN patient. National costs of health care reimbursement in the general population were obtained from NHF open data [10]. Demographic data on the Polish population from the Polish Department of Statistics was used to calculate the costs per citizen [11]. Medium yearly costs of heath per polish citizen were compared with the cost of HPN patients. Data were collected in polish currency (zloty) and calculated in euro based on medium courses of polish zloty/ euro from the polish national bank [12]. 

### 2.3. Statistical Analysis

Qualitative variables were shown as counts with percentages. We built linear regression models to verify possible trends across years. During this process, we treated our data set as a sample rather than a population. Results from linear regression were expressed as beta coefficients with 95% confidence intervals (CIs). The 2-tailed tests were carried out at a significance level of 0.05. Statistical analysis was performed using the R statistical package (version 3.6.3.).

## 3. Results

### 3.1. Prevalence and Age Distribution 

**Figure 1:**
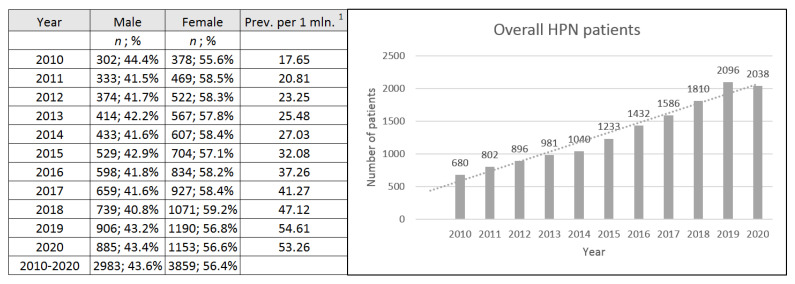
Prevalence of home parenteral nutrition in Poland. ^1^—Prevalence of HPN patients per 1 million citizens.

6842 patients (56.4% F, 43.6% M) were treated with HPN in the observation period. A 2.99-fold increase in patients was noted between 2010 and 2020 with a *p*-trend < 0.001 (beta coefficient: 148.3, 95%CI: 127.8–168.7); however, a noticeable decrease was observed in 2020. Trend prognosis for 2020 was 2082 with 95%CI: (1925–2239) vs. 2038 in real data (Figure 1).

**Figure 2:**
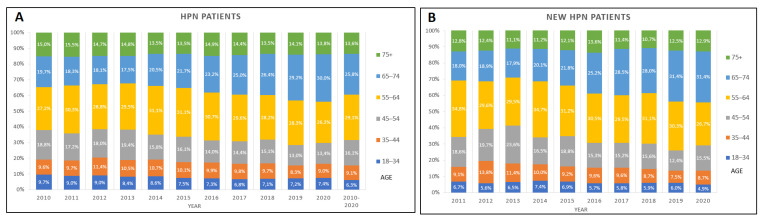
Age distribution. (**A**)—HPN patients, (**B**)—new qualifications.

54.86% of patients were 55–74 years old (29.09%—55–64, 25.76%—65–75). From 2010 to 2020 there was a significant decrease in the percentage of patients between 18–34 (estimate −0.264, with 95%CI: −0.358: −0.169, *p*-trend < 0.001) and between 45–54 (estimate −0.582, with 95%CI: −0.813: −0.351, *p*-trend < 0.001). Significant increase in the percentage of patients between 65–74 (estimate 1.249, with 95%CI: 0.891: 1.607, *p*-trend < 0.001) and patients over 75 years old (estimate −0.129, with 95%CI: −0.253: −0.006, *p*-trend- 0.042) (Figure 2). 453,750 patient days of HPN were recorded in 2020 which gives a 2.9-fold increase in comparison to 2010 (Table 2).

There were 6185 new qualifications for HPN (data available from 2011 to 2020). Trend analysis showed increase in the percentage of new patients between 65–74 (estimate 1.740, with 95%CI: 1.377: 2.103, *p*-trend < 0.001). Significant decrease was observed in new patients between 35–44 (estimate −0.381, with 95%CI: −0.732: −0.030, *p*-trend < 0.037) and between 45–54 (estimate −0.767, with 95%CI: −1.331: −0.204, *p*-trend < 0.014) (Figure 2). Detailed statistical information in (Supplementary Materials Table S1). 

### 3.2. Indications for HPN

Malnutrition, intestinal malabsorption/other intestinal diseases and postprocedural disorders of the gastrointestinal tract (GI) were the top 3 primary diagnoses for HPN. 1519 patients (17.3% of primary and 23.16% of secondary diagnoses) were qualified due to cancer diagnosis mostly gastric, ovarian, colon and pancreatic cancer (Table 1). 

### 3.3. The Costs of HPN

The cost of adult HPN reimbursement from 2010–2020 was more than € 146 million and the total cost of health-related reimbursement of HPN patients was nearly € 242 million. The percentage of HPN costs in total health care cost of HPN patients was on average 61%. HPN and total health cost reimbursement increase was 2.6 and 2.57- fold respectively with an increasing trend (*p*-trend < 0.001). The number of patient days increased by 2.9-fold in the observation period. The cost of HPN per patient treated during following years was decreasing (estimate −209.478, with 95%CI: −303.436: −115.521, *p*-trend < 0.001) as well as the total costs of health care per HPN patient (estimate −184.008, with 95%CI: −296.679: −71.337, *p*-trend < 0.005). The mean cost of health per citizen in Poland was on average 36.8 times lower than health care costs per HPN patients (Table 2). 

### 3.4. Hospitalizations of HPN Patients

4301 patients were hospitalized, and 18,733 hospital stays were recorded (Table 3). 9819 primary, 18,388 secondary diseases and 56,870 medical procedures were reported. Cancer, infection, malnutrition and GI symptoms were the most common reasons for the hospital stay. Cancer was reported as the main or comorbid disease in 5351 cases (Table 4). Diagnostic procedures and oncological treatment (mainly chemotherapy) were the most common procedures (Table 5). 

### 3.5. Length of Nutrition and Short-Term Survival

The analysis included patients qualified for HPN in the observation time. Therefore, the maximal length of nutrition was 11 years (observation time 2010–2020) (Table 6). The rate of patients with a maximal length of HPN of 5 months in 2010 was 54.9% and was growing up to 78.1% in 2020 (Table 7).

Overall death rate during the first year of nutrition was 0.59 with a significant increase in the observation period trend *p*-trend < 0.004. An increase in the death rate was observed in patients between 55–64 (estimate 1.174 with 95%CI: 0.081: 2.266, *p*-trend < 0.039) and above 75 years old (estimate 1.629, with 95%CI: 0.208: 3.050, *p*-trend < 0.030). Trend analysis showed no statistically significant differences in the death rates in every other group (Table 8).

## 4. Discussion

Long-term observations of the HPN population give a wide perspective on the evolution and possible future development of the outpatient treatment of IF. The prevalence of adult HPN in Poland grew from 17.65 patients per 1 min. of citizens in 2010 to 53.26 in 2020. A nearly 3-fold increase in a total number of HPN patients was confirmed in the trend analysis. 2020 was an exceptional year with a decrease in the total number of HPN patients. A projected number from the trend analysis was 2082 and real data showed 2038 HPN patients. We assume that this could be a consequence of the Covid-19 pandemic, however continuous observations in the following years are needed to confirm this. 

There are only a few studies on HPN prevalence with trending analysis. However, the growing prevalence in other countries can be estimated from the point prevalence data presented in several studies. The first European multicenter report of the ESPEN Home Artificial Nutrition (HAN) group published in 1996 showed the prevalence of 4.6 to 12.2 patients per million citizens in Europe and 0.36 in Poland [13]. An Italian study from 2005 showed 31.7 HPN patients per million citizens [14] and data from Campalila region confirmed a 96% increase in patients on HPN (156 on HPN in April 2005 and 306 in 2012) [15]. US analysis for 2013 showed 79 HPN patients per million citizens [16]. Data updated from national registries come from Spain and UK. NADYA-SENPE group published reports from the Spanish HAN registry from 2013, 2016 and 2018 with a prevalence of 4.22, 6.16 and 6.61 per million citizens [17,18,19]. In the UK, prevalence of HPN together with home intravenous fluids (HIVF) in 2015 was 40 per million citizens. A more than 2-fold increase in period prevalence was observed from 2005 to 2015 [20]. 

We additionally described the trends in the age distribution showing that the propotion of younger patients qualified for HPN are decreasing. Those trends are noticeable in the age range between 18–54 years old. On the other hand, the percentage of patients over 65 has increased. Similar tendencies are shown among new qualifications with increasing trends in the 65–74 age group and a decrease in young patients 35–54. This confirms the hypothesis of the aging HPN population. Data from the British Artificial Nutrition Survey (BANS) report showed that the majority of HPN patients were under 71 years old (81.5%) and two-thirds were between 41 and 70. However, in the observation period between 2005 and 2015, the age distribution didn’t change significantly in the UK [20]. In the previously mentioned study of the ESPEN HAN group 28% of patients in Europe were older than 61 years when they started HPN and 10%—were more than 70 years old [13]. The effect of the changing age distribution on the outcomes of HPN patients’ needs to be analyzed in future studies. A retrospective study from Canada showed that older patients on HPN have higher 2-year mortality but a lower risk of catheter-related bloodstream infections [21].

The duration of HPN in our study was calculated as the time between the first and last reported HPN in the base of NHF. This is a limitation of the analysis, and the rates of long-term nutrition may be underrepresented. Some patients might have been on HPN for a longer time exciding the observation period of the study. However, the duration of HPN was mostly shorter than one year (71.14%). We additionally showed an increase in the rates of short term (maximal of 5 months) HPN and the risk of death during the first year after qualification for HPN was 0.59 and increased significantly in the observation period (estimate 1.210, with 95%CI: 0.504: 1.881, *p*-trend < 0.030). We were not able to compare the survival between the primary diagnosis. However, growing numbers of new qualifications among oncological patients may explain the trends in the risk of death and short term HPN.

Malnutrition was reported most commonly in HPN patients (34% as primary and 47% as secondary diagnosis) following intestinal malabsorption or other intestinal diseases. Advanced cancer was diagnosed as a primary disease in 17.36% of cases and secondary disease 23.1%. More than a third of patients with mechanical bowel obstruction due to cancer were gastric cancer patients following ovarian and colon cancer in 17.8% and 12.3% respectively. Having the methodology of the study in mind, it is more difficult to estimate the rates of other diseases. For instance, reported ICD-10 of Inflammatory Bowel Disease (IBD) were only for 2.11% of HPN but we may assume that some of the patients with malabsorption, malnutrition or postprocedural disorders might be also Crohn or ulcerative colitis patients. Moreover, data on the indications for HPN were provided for the whole observation period with no specified information for each year, therefore, an evolution of indications was not possible to determine. Nevertheless, high rates of oncological patients are in line with other data from the literature. In the previously mentioned NADYA study, the most frequent diagnosis in adults was “palliative cancer” (25.6%) and the cancer diagnosis concerned 44% of patients: either after radical treatment or with post-radiation enteritis [22]. This was confirmed in other data from Spain [23] and Switzerland [24] (49.5% and 57.4% cancer patients). BANS report showed that cancer was the main reason for IF in HPN for one in four new registrations, however, Crohn disease remained a leading diagnosis of 14% new and 21% of HPN patients in point prevalence analysis from 2015 [20]. Data from the Canadian registry comparing two periods 2011–2014 and 2005–2008 also showed increased rates of cancer patients (37.9% vs. 16.7%) and a reduction in short bowel syndrome patients (32% vs. 65.5%). The median age of HPN patients increased from 52 to 57 in this study confirming our data on the trends in age distribution [25]. 

The reimbursement of HPN in Poland started in 1998. It covers the costs of HPN provision, monitoring and the support of a specialized nutrition team. In our study, we analyzed the costs reported by the NHF. The average cost of HPN per patient (yearly) in the observational period was € 10,251.2. NHF reimbursement of HPN has not changed in the observation time and is between € 45–55 for a patient day (depending on the zloty/euro currency conversion rate for the following years). Total costs of health care per patient (in the HPN population) were € 16,743 in comparison with the average cost of health care from 2010–2020 in general polish population—€ 486.6. This included all costs of hospital stays and treatment procedures (like surgery or oncological treatment), ambulatory care and rehabilitation, drugs reimbursement, diagnostic procedures. Since we analyzed data from NHF, we were not able to calculate the costs covered by the patients (like traveling to the hospital, caregiver, job loss or the need to limit professional work). Interestingly, we found that the cost of HPN and total costs of health care per HPN patient decreased during the observation time. NHF provided data for the general population of HPN patients and the cost per patient was calculated using data on the total number of individual patients in the following years. Consequently, a significant decrease in the cost of HPN and total costs of health care per HPN patient in the observation period can be explained by a growing rate of short-term HPN described in Table 7. Although there are studies showing the cost of HPN of € 8000 per patient per year [26] most calculated from € 14,000 up to € 77,000 in Europe [9,27,28,29]. The costs in Spain were 13,363.53 per patient per year and € 124.02 per patient per day. From overall costs, the HPN provision accounted for the highest percentage (74.25%), followed by management of complications (21.85%) with the CRBSI rate of 2.03 per 1000 HPN days and, outpatient monitoring (2.23%). HPN bags were the most expensive element in the HPN provision [26]. Other studies confirmed this thesis [30,31,32]. UK study—£ 30,000–40,000 per patient per year for the self-cared patient and £ 55,000–65,000 per patient per year with nursing support [33]. Data from the US show cost of $ 64,000 per patient per year [34]. High costs of total health care observed in the population of HPN patients in comparison with the general population may relate to comorbidity, advanced medical therapies (surgery, oncological procedures) and the treatment of the complications. Additional costs of health care of patients with central line-associated bloodstream infections, catheter-associated urinary tract Infections or surgical site Infections were estimated: $48,108, $13,793 and $28,219 [35]. Additional analysis of hospitalizations of HPN patients from our study confirms this thesis. For nearly 40% of patients the oncological disease was a primary or secondary cause of hospitalization. For more than 20% of patients the hospitalizations involved procedures of the oncological treatment (mostly chemotherapy). Infectious complications were reported in 11% as primary and 8.7% as a secondary diagnosis in hospitalized HPN patients.

### Limitations of the Study

We are aware that data analysis from the NHF has some limitations. Firstly, the analysis of the indications for HPN and hospitalizations was based on ICD-10. This method may be more useful for oncological patients however it may not always reflect the epidemiology for benign patients. Malnutrition, intestinal malabsorption or postprocedural disorders may indicate many more specific indications like IBDs. Indications and hospitalization were not analyzed annually therefore we were not able to address the trends in those areas. Additionally, cost analysis based on the data from NHF reported annually. Data on costs per HPN patient or per polish citizen needed to be calculated based on the provided data of HPN population and general statistics of polish population. 

## 5. Conclusions

Long-term observations show that the prevalence of HPN is growing. Trend analysis showed more advanced age in the HPN population with a growing rate of patients over 65 years old. Oncological patients become dominant in the HPN population. Consequently, rates of short-term HPN with higher mortality are increasing. Total costs of health care in the HPN population are high and may relate to advanced treatment and complications. Hospitalizations of HPN were most often due to oncological treatment, infections, GI symptoms and malnutrition. 

## Figures and Tables

**Table 1:** Main and secondary diagnosis.

**Diagnosis.**	**Primary**	**Secondary**		**GI obstruction (Cancer)**	***n*; %**
				Gastric cancer	528; 34.74
	***n*; %**	***n*; %**		Ovarian cancer	271; 17.83
Malnutrition	2632; 34.28	381; 47.45		Colon cancer	187; 12.3
				Pancreatic cancer	126; 8.29
Intestinal malabsorption/other intestinal diseases	1567; 20.41	62; 7.72		Other/non specified/disseminated	106; 6.97
				Rectal cancer	76; 5.00
Postprocedural disorders of digestive system	1506; 19.61	70; 8.72		Esophageal cancer	72; 4.74
				Uterine cancer	50; 3.29
GI obstruction (cancer)	1333; 17.36	186; 23.16		Liver/ biliary/gallbladder cancer	25; 1.64
				Small intestine cancer	22; 1.45
Other/Not specified	204; 2.66	65; 8.09		Lungs and respiratory tract cancer	20; 1.32
				Urinary blader cancer	17; 1.12
GI obstruction	198; 2.58	28; 3.49		Mammary cancer	14; 0.92
				Lyphoma	6; 0.39
IBD	162; 2.11	11; 1.37		**IBD**	
				Crohn disease	130; 75.14
Vascular disorders of intestine	77; 1	0; 0		Other intestinal infmation	35; 20.23
				Colitis ulcerosa	8; 4.62

IBD—Inflammatory Bowel Disease.

**Table 2:** Costs of HPN and total health care in Poland–NHF data.

Year.	Costs of HPN	Patient Days	Costs of Health Care (HPN Patients)	Nutrition in Health Care % ^1^	Costs of HPN per Patient	Health Care per HPN Patient	NHF Health Costs per Citizen ^2^	NHF Costs of Health Care ^3^
2010	7,829,635.9	156,350	12,719,715.6	61.6	11,514.2	18,705.5	391.4	15,081,321,298.0
2011	8,644,043.1	178,050	13,683,367.3	63.2	10,778.1	17,061.6	386.3	14,886,623,215.8
2012	9,867,620.8	206,850	15,277,280.9	64.6	11,013.0	17,050.5	404.0	15,567,582,110.9
2013	10,448,827.9	219,300	16,383,171.3	63.8	10,651.2	16,700.5	414.5	15,958,183,724.0
2014	11,346,517.1	237,250	17,513,570.4	64.8	10,910.1	16,840.0	423.6	16,300,111,193.5
2015	12,822,785.4	267,650	20,881,042.5	61.4	10,399.7	16,935.2	445.5	17,122,412,686.2
2016	13,510,350.2	294,350	22,465,231.6	60.1	9,434.6	15,688.0	448.3	17,227,868,912.7
2017	15,361,876.8	327,400	26,334,310.9	58.3	9,685.9	16,604.2	496.6	19,086,788,739.0
2018	17,077,692.0	363,900	29,659,525.5	57.6	9,435.2	16,386.5	524.4	20,142,396,696.2
2019	18,707,546.3	402,100	33,890,853.3	55.2	8,925.4	16,169.3	578.1	22,190,154,461.7
2020	20,412,065.0	453,750	32,686,295.2	62.4	10,015.7	16,038.4	641.4	24,541,542,319.9

All costs in Euro, calculated from polish currency (zloty) according to the current courses of polish zloty/ euro from the Polish National Bank. ^1^—Costs of HPN /Costs of health care (HPN patients) × 100%. ^2^—NHF health costs per citizen calculated for the general population of Poland in following years from the Polish Department of Statistics [11]. ^3^—Total costs of health care in Poland reported by NHF.

**Table 3:** Hospitalizations between 2011–2020.

Year	2011	2012	2013	2014	2015	2016	2017	2018	2019	2011–2019
Patients ^1^	257	291	301	340	459	529	603	680	841	4301
Hospitalizations	901	1072	1129	1182	2044	2132	2891	3173	4209	18,733

^1^—Number of unique patients hospitalized.

**Table 4:** Diagnosis during hospitalizations.

Diagnosis	Main	Comorbid
	*n*; %	*n*; %
Other	3209; 32.7	3823; 20.8
Cancer	1990; 20.3	3361; 18.3
Infection/sepsis	1142; 11.6	1604; 8.7
Malnutrition	893; 9.1	1667; 9.1
GI symptoms/diseases	636; 6.5	2718; 14.8
Surgical complications	424; 4.3	755; 4.1
Respiratory track diseases	360; 3.7	869; 4.7
Haematological diseases	326; 3.3	1306; 7.1
Renal disease	296; 3	604; 3.3
Cardiologic disease	276; 2.8	1179; 6.4
Thrombotic and vascular	163; 1.7	312; 1.7
Liver diseases	81; 0.8	185; 1
Dysphagia	23; 0.2	5; 0

**Table 5:** Procedures during hospitalizations.

Procedures	*n*; %
Other	20,164; 35.46
Diagnostic procedures	19,305; 33.95
Oncological treatment	12,248; 21.54
Surgical treatment	3377; 5.94
Intensive care/anasthesia	1776; 3.12
All	56,870

**Table 6:** HPN length.

HPN Length (Years)	*n*; %
0–0.5	3926; 57.7
0.5–1	911; 13.4
1–2	806; 11.9
2–3	322; 4.7
3–4	207; 3
4–5	131; 1.9
5–11	496; 7.3

**Table 7:** Short term HPN.

Year	2011	2012	2013	2014	2015	2016	2017	2018	2019	2020
Short HPN ^1^	180	195	226	223	302	376	406	512	609	702
New patients ^2^	328	355	386	418	554	646	722	850	1027	899
Rate of short HPN in %	54.9	54.9	58.5	53.3	54.5	58.2	56.2	60.2	59.3	78.1

^1^—Short term HPN—Number of new patents in following years with a maximal length of HPN of 5 months. ^2^—All new patients in Poland in the following years.

**Table 8:** HPN death rate during first year—Age distribution.

	Age Distribution						
Year	18–34	35–44	45–54	55–64	65–74	75+	All
2011	0.00	0.43	0.57	0.63	0.54	0.55	0.53
2012	0.44	0.51	0.47	0.58	0.60	0.55	0.54
2013	0.36	0.39	0.69	0.57	0.55	0.58	0.56
2014	0.19	0.45	0.64	0.59	0.52	0.62	0.54
2015	0.38	0.45	0.57	0.58	0.57	0.59	0.55
2016	0.32	0.44	0.65	0.66	0.70	0.61	0.62
2017	0.31	0.41	0.63	0.69	0.56	0.54	0.57
2018	0.22	0.65	0.61	0.68	0.67	0.67	0.64
2019	0.23	0.55	0.65	0.66	0.62	0.73	0.62
2011–2019	0.31	0.49	0.61	0.64	0.61	0.63	0.59
p *	0.802	0.176	0.269	0.039	0.12	0.03	0.004

* trend *p*-value.

## Supplementary Materials

The following are available online at https://www.mdpi.com/article/10.3390/nu13103465/s1, Table S1. HPN—Age distribution, trend analysis.
