# Supplementary material for: Trend Observations in Home Parenteral Nutrition. Prevalence, Hospitalizations and Costs: Results from a Nationwide Analysis of Health Care Provider Data
Source: Nutrients. 2021 Sep 29;13(10):3465. doi: 10.3390/nu13103465 (PMC8539115; doi:10.3390/nu13103465)
Supplement: Supplementary file 1 [file nutrients-13-03465-s001.zip › nutrients-1376345-supplementary.pdf]

Supplementary material

Table S9. HPN – Age distribution, trend analysis

	estimate		CI[3] lower estimate		CI upper estimate		Trend p-value	
Age	All[1]	New[2]	All	New	All	New	All	New
18–34	-0.264	-0.136	-0.358	-0.297	-0.169	0.025	< 0.001	0.088
35–44	-0.143	-0.381	-0.298	-0.732	0.012	-0.030	0.066	0.037
45–54	-0.582	-0.767	-0.813	-1.331	-0.351	-0.204	< 0.001	0.014
55–64	-0.132	-0.462	-0.477	-0.996	0.214	0.072	0.411	0.081
65–74	1.249	1.740	0.891	1.377	1.607	2.103	< 0.001	< 0.001
75+	-0.129	0.006	-0.253	-0.244	-0.006	0.257	0.042	0.954

1- All HPN patients treated during the observation period (2010-2020)
2- New qualifications for HPN (2011-2020)
3- Confidence interval
